# Effect of Rising Time on AC Stress-Induced Performance Degradation in a-ITGZO Thin-Film Transistors

**DOI:** 10.3390/nano15120880

**Published:** 2025-06-07

**Authors:** Mingu Kang, Kyoungah Cho, Sangsig Kim

**Affiliations:** Department of Electrical Engineering, Korea University, 145 Anam-ro, Seongbuk-gu, Seoul 02841, Republic of Korea; 97mgkk@korea.ac.kr

**Keywords:** amorphous indium–tin–gallium–zinc-oxide, AC bias stress, TCAD simulation, thin-film transistor

## Abstract

In this study, we investigate the impact of rising time on alternating current (AC) stress-induced degradation in amorphous indium–tin–gallium–zinc oxide (a-ITGZO) TFTs through both experiments and simulations. When AC bias stresses with rising and falling times (*t*_r-f_) of 400 ns, 200 ns, and 100 ns were applied to the a-ITGZO TFTs, the threshold voltage (*V*_TH_) shifted positively by 0.97 V, 2.68 V, and 2.83 V, respectively. These experimental results align with a stretched exponential model, which attributes the *V*_TH_ to electron trapping in bulk dielectric states or at interface traps. The simulation results further validate the stretched exponential model by illustrating the potential distribution across the dielectric and channel layers as a function of *t*_r-f_ and the density of states in the a-ITGZO TFT.

## 1. Introduction

Recently, oxide thin-film transistors (TFTs) have been integrated into low-temperature polycrystalline silicon and oxide (LTPO) TFTs, which are emerging as a next-generation backplane technology for portable electronics utilizing active matrix organic light-emitting diode (AMOLED) displays [1,2,3,4,5,6,7]. These displays demand not only high resolution and brightness, but also low power consumption and a fast response time. LTPO technology is to combine the high mobility of poly-Si TFTS for driving and the low leakage characteristic and uniformity of oxide TFTs for pixel switching. To enable high-performance LTPO TFTs in pixel circuits, the reliability of oxide TFTs under alternating current (AC) stress is critical, as they are inevitably exposed to AC stress in integrated gate driver circuits. This AC instability is a major contributor to the performance degradation of LTPO TFTs.

To date, research on the degradation of electrical characteristics in oxide TFTs under AC bias stress has primarily focused on the interface between the channel and the gate dielectric [8,9,10,11]. However, performance degradation in TFTs is generally attributed to both bulk traps within the gate dielectric and interface traps at the dielectric/channel boundary [12,13]. Despite this, limited attention has been given to the role of bulk traps in the gate dielectric under AC bias conditions. In addition, most research has been conducted on the AC bias stress applied to the drain electrode, not to the gate electrode [14,15,16]. However, the AC bias stress applied to the gate electrode can profoundly affect the performance of TFTs, since the repetitive gate voltage pulse can accelerate trap generation and electron trapping within the gate dielectric and at the dielectric/channel interface.

In this study, we investigated the electrical characteristics of oxide TFTs under AC bias stress by considering both bulk traps within the gate dielectric and interface traps between the gate dielectric and the channel. Among the various AC parameters, the rising and falling times are fundamentally important for high-speed electronics [17]. In AMOLED driving circuits designed for high refresh rates, the gate electrodes of TFTs suffer from AC bias stress from high-frequency pulses. The rising edge of AC pulses brings about rapid changes in the potential across the gate dielectric and channel so that significant transient stress is applied to the TFTs. Thus, the rising edge of AC pulses is responsible for the performance degradation of TFTs. Hence, understanding the degradation mechanisms associated with AC bias stress under fast-rising conditions is essential for improving the reliability of TFTs in practical applications. Nevertheless, to date, there is little research on the degradation mechanisms in oxide TFTs from the rising edge of AC pulses. Therefore, we specifically examined the effect of rising time on AC stress-induced performance degradation in amorphous indium–tin–gallium–zinc oxide (a-ITGZO) TFTs through a combination of experimental analysis and simulation.

## 2. Materials and Methods

In this study, top-gate coplanar a-ITGZO TFTs with a split channel structure were fabricated on SiO_2_/p-type Si substrates. The split structure of the channel minimizes the parasitic series resistance so that it can enhance the field-effect mobility (μ_FE_) and the on/off current ratio [18]. A 50 nm-thick a-ITGZO channel layer was deposited from an ITGZO target (In_2_O_3_:SnO_2_:Ga_2_O_3_:ZnO = 0.8:0.2:1:1 mol%, iTASCO, Korea) using radiofrequency (RF) magnetron sputtering (SCIEN Tech, Korea) under a gas mixture of Ar/O_2_ (47:30 sccm) at an RF power of 120 W and a working pressure of 1 mTorr. The channel length and width were 100 µm and 50 µm, respectively. The channel was formed by the ten split channels with a width of 5 μm, and the separation between the channels was 25 μm.

**Figure 1:**
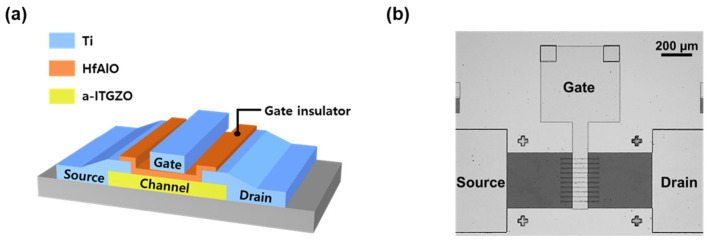
(**a**) Schematic and (**b**) optical microscope image of the fabricated a-ITGZO TFT.

A 60 nm-thick HfAlO gate dielectric layer was deposited by atomic layer deposition (ALD) system (Lucida D100, NCD Technology, Korea) at 150 °C. During deposition, H_2_O was used as the oxidant, while tetrakis (ethylmethylamino) hafnium and trimethylaluminum served as the precursors for Hf and Al, respectively. The source, drain, and gate electrodes were formed via the thermal evaporation (SCIEN Tech, Korea) of Ti. Figure 1 presents a schematic (Figure 1a) and an optical microscope image (Figure 1b) of the fabricated a-ITGZO TFT.

The thicknesses of the a-ITGZO and HfAlO layers were measured using X-ray reflectometry (D8 Discover Plus, Bruker, MA, USA), and the capacitance of the gate dielectric was measured using an LCR meter (HP4285A, Agilent Technologies, Santa Clara, CA, USA). The I–V characteristics were evaluated using a semiconductor parameter analyzer (HP4155C, Agilent Technologies, Santa Clara, CA, USA), and AC bias stress was applied using an SMU/pulse generator (16440A, Agilent Technologies, Santa Clara, CA, USA). The electrical characteristics were measured under both DC and AC bias stress conditions in ambient air and inside a dark box.

A unipolar AC pulse (0 to 15 V) with a frequency of 500 kHz was applied to the gate electrode. The pulse had a duty ratio of 50% with equal rising and falling times. To examine the effect of the pulse rising–falling time (*t*_r-f_) on the electrical characteristics of a-ITGZO TFTs under AC bias stress, we applied AC pulses with *t*_r-f_ values of 400, 200, and 100 ns. All simulations in this study were conducted using the Silvaco Victory TCAD device simulator (version 1.14.1.R).

## 3. Results and Discussions

**Figure 2:**
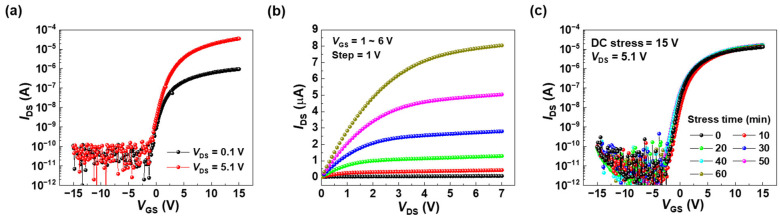
(**a**) Transfer and (**b**) output characteristics of the a-ITGZO TFT. (**c**) Time evolution of the transfer curves under a DC bias stress of 15 V.

Figure 2 presents the transfer characteristics of the a-ITGZO TFT measured at drain-to-source voltages of 0.1 V and 5.1 V (Figure 2a), and its corresponding output characteristics (Figure 2b). The *μ*_FE_ in the linear region and the subthreshold swing (*SS*) are calculated using the following equations:

(1) $$I_{DS}=\frac{C_{ox}\mu_{FE}}{2}\frac{W}{L}[2\left(V_{GS}-V_{TH}\right)V_{DS}-V_{DS}^{2}],$$

(2) $$SS=\frac{{\partial V}_{GS}}{\partial \left(\log I_{DS}\right)}$$

where *I*_DS_ is the drain-to-source current, *C*_ox_ (=119 nF/cm^2^) is the gate dielectric capacitance per unit area, *W*/*L* (50/100 μm) is the channel width-to-length ratio, and *V*_TH_ is the threshold voltage. *V*_TH_ was determined using the constant current method, using the *V*_GS_ corresponding to an *I*_DS_ of *W*/*L* × 10 nA at a *V*_DS_ of 5.1 V. The *μ*_FE_, *SS*, and *V*_TH_ values of the a-ITGZO TFTs were 16.8 ± 3.4 cm^2^/V∙s, 447 ± 40 mV/decade, and –0.5 ± 0.3 V, respectively. Figure 2c shows the time evolution of the transfer curves for the a-ITGZO TFT under a DC bias stress of 15 V. The time evolution reveals that the on-current, *SS*, and threshold voltage shift (Δ*V*_TH_) are slightly changed even after the DC bias stress for 60 min; the on-current is changed from 13.0 μA to 16.6 μA, the *SS* from 670 mV/dec to 686 mV/dec, and the *V*_TH_ from −0.6 V to −0.9 V. Considering that DC bias stress does not significantly change the performance of the a-ITGZO TFTs, this suggests that electron trapping rarely occurs under DC bias conditions.

**Figure 3:**
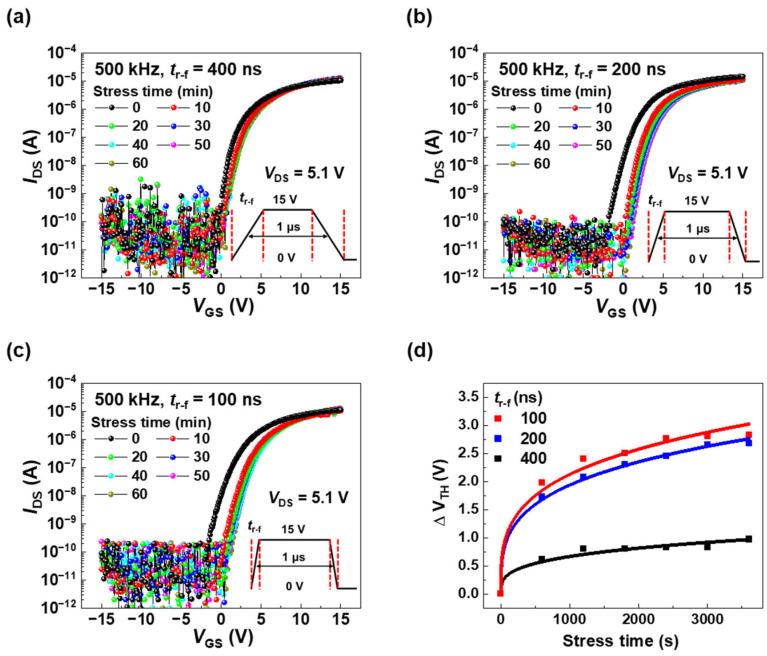
Transfer curves of the a-ITGZO TFTs under AC pulse conditions with *t*_r-f_ of (**a**) 400 ns, (**b**) 200 ns, and (**c**) 100 ns. (**d**) Time dependence of Δ*V*_TH_ under AC bias stress. Scattered points and solid lines represent the experimental Δ*V*_TH_ values and the corresponding values calculated using the stretched exponential model, respectively.

On the other hand, the AC bias stress significantly affects the *V*_TH_ of the a-ITGZO TFTs, depending on the *t*_r-f_ of the AC pulse, as shown in Figure 3, which presents the time evolution of the transfer curves for *t*_r-f_ values of 400 (Figure 3a), 200 (Figure 3b), and 100 ns (Figure 3c). Nevertheless, the on-current and *SS* of the a-ITGZO TFTs under AC bias stress showed only slight changes compared to those of the pristine devices. The degradation of the *SS* is expressed via Δ*SS* before and after applying AC bias stress. For *t*_r-f_ values of 400 ns, 200 ns, and 100 ns, the Δ*SS* values are 28 mV/dec (from 398 mV/dec to 426 mV/dec), 51 mV/dec (from 550 mV/dec to 601 mV/dec), and 62 mV/dec (from 527 mV/dec to 589 mV/dec), respectively, indicating that the *SS* is significantly degraded as *t*_r-f_ becomes shorter. Nevertheless, the AC bias stress has a considerable impact on Δ*V*_TH_, as shown in Figure 3d, rather than on the degradation of the *SS*. In addition, the AC bias stress has relatively little impact on the magnitude of the on-current. The on-current changes from 10.4 μA to 12.6 μA for a *t*_r-f_ value of 400 ns, from 14.3 μA to 10.7 μA for a *t*_r-f_ value of 200 ns, and from 11.3 μA to 12.5 μA for a *t*_r-f_ value of 100 ns. On the contrary, the AC bias stress has a major impact on the *SS* and on-current rather than on Δ*V*_TH_ when the AC bias has a low level of −20 V and a high level of 20 V [19,20]. In this study, the AC bias has a low level of 0 V and a high level of 15 V, which is close to the real-world situation in which the low level of a clock pulse is 0 V in display circuits. Under AC bias stresses with *t*_r-f_ values of 400 ns, 200 ns, and 100 ns, the Δ*V*_TH_ values are 0.97 V (from 0.57 V to 1.54 V), 2.68 V (from −0.44 V to 2.24 V), and 2.83 V (from −0.38 V to 2.45 V), respectively. This indicates that a larger Δ*V*_TH_ is induced when AC pulses with faster *t*_r-f_ are applied to the a-ITGZO TFTs. Figure 3d shows the time dependence of Δ*V*_TH_ under AC bias stress with *t*_r-f_ values of 100, 200, and 400 ns. Here, the scattered points and solid lines represent the experimental Δ*V*_TH_ values and those calculated from a stretched exponential model, respectively. Considering the coefficient of determination (R^2^) that assesses how well a model fits the data, the data are well fitted with a stretched exponential equation; the values of R^2^ for 100 ns, 200 ns, and 400 ns are 0.98517, 0.99788, and 0.96733, respectively. The experimental Δ*V*_TH_ values align well with the model, which explains Δ*V*_TH_ as a result of electron trapping in the bulk states of the gate dielectric or at interface traps between the channel and dielectric layers [21,22].

The equation for the stretched exponential model is given as follows:

(3) $$∆V_{TH}\left(t\right)={(V}_{G}-V_{TH0})[1-\exp\{-{\left(\frac{t}{\tau }\right)}^{\beta }\}],$$

where *V*_G_ is an applied gate bias, *V*_TH0_ is the initial threshold voltage_,_ τ is the characteristic trapping time of carriers, and *β* is the stretched exponential exponent (in this study, *β* is 0.3) [23,24]. Furthermore, τ is expressed as τ = τ_0_ exp(*E*_τ_/kT), where τ_0_ is the thermal prefactor for emission over the barrier, and *E*_τ_ is the average effective energy barrier that electrons in the channel must overcome to enter the insulator [25]. Thus, the τ value reflects the height of *E*_τ_; a higher *E*_τ_ corresponds to a longer time required to overcome the barrier.

In this study, the τ values obtained from the stretched exponential equation are 6.1 × 10^5^, 8.1 × 10^5^, and 2.6 × 10^7^ s for *t*_r-f_ values of 100, 200, and 400 ns, respectively. The difference in τ values between 400 ns and 200 ns is significantly larger than that between 200 ns and 100 ns, indicating that the nonlinearity in Δ*V*_TH_ is deeply concerned with the difference in τ values. As the rising time is faster, the *E*_τ_ for electron trapping is lower, so that the change in Δ*V*_TH_ becomes greater. These τ values fall within the range reported in other studies on charge trapping mechanisms under bias stress [9,22,23,24,25]. The shorter trapping time observed at *t*_r-f_ = 100 ns compared to 400 ns implies that *E*_τ_ is lower at faster rising times, resulting in a significantly larger Δ*V*_TH_, which agrees with our experimental findings.

**Figure 4:**
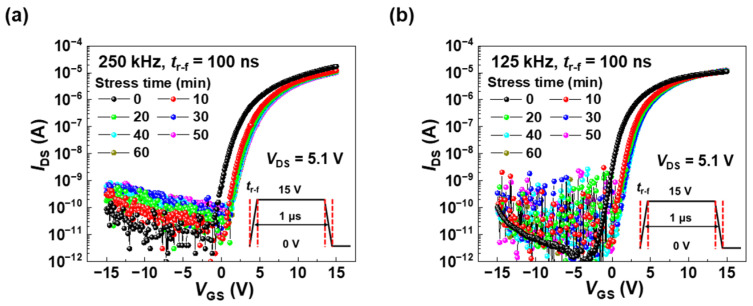
Transfer curves of a-ITGZO TFTs under AC pulse conditions with frequencies of (**a**) 250 kHz and (**b**) 125 kHz.

Among the AC bias conditions, the pulse frequency along with the rising time has been known to be one of key parameters in the performance degradation of TFTs [26,27]. Hence, we examined the effect of the pulse frequency on the electrical characteristics of a-ITGZO TFTs at *t*_r-f_ of 100 ns, which was the rising time resulting in the most pronounced degradation in *V*_TH_, as shown in Figure 3. Figure 4 represents the transfer curves under AC stress with frequencies of 250 kHz (Figure 4a) and 125 kHz (Figure 4b), indicating that Δ*V*_TH_ increases with the pulse frequency; 1.98 V at 125 kHz, 2.12 V at 250 kHz, and 2.83 V at 500 kHz (referred to Figure 3). The change in Δ*V*_TH_ is deeply concerned with the number of AC pulses applied to the TFT. As the frequency is higher, the number of AC pulses applied to the TFT increases for a certain period. Thus, the gate dielectric and the interface between the channel and dielectric layers experience more frequent voltage transitions, increasing the probability of electron injection into bulk trap states within the gate dielectric or into interface trap states.

**Figure 5:**
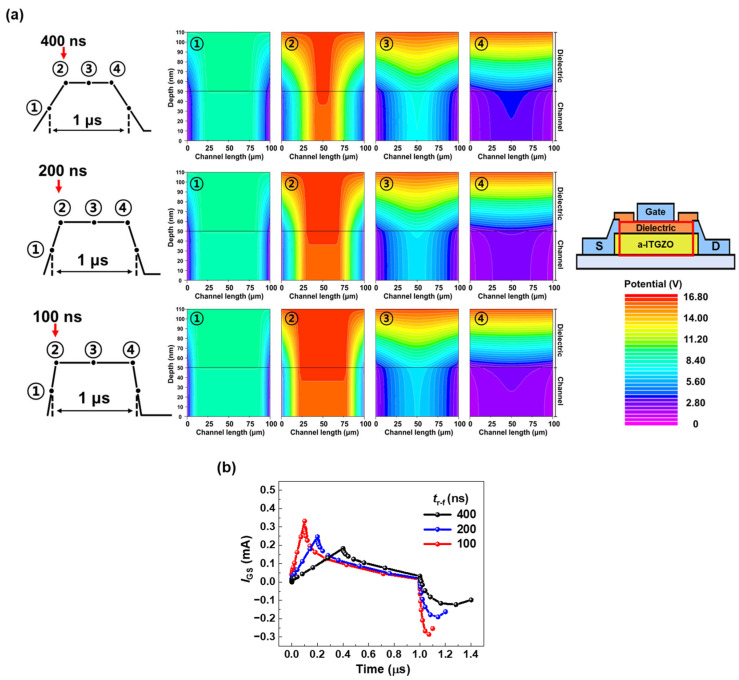
(**a**) Potential contours of the dielectric and channel layers when the AC pulse with *t*_r-f_ of 400 ns, 200 ns, and 100 ns is applied. (**b**) Gate leakage current (*I*_GS_) over one cycle of AC pulses with *t*_r-f_ of 400 ns, 200 ns, and 100 ns.

As mentioned above, according to the stretched exponential model, Δ*V*_TH_ arises from electron trapping in the bulk states of the gate dielectric or at interface traps. However, most previous studies on Δ*V*_TH_ have focused primarily on electron trapping at interface traps [28,29,30]. Therefore, in this study, to verify that electron trapping in the bulk dielectric is responsible for Δ*V*_TH_, we investigated the transient characteristics through the potential distribution across the dielectric and channel layers when applying AC bias stress. Figure 5a presents the potential contours extracted at four specific time points for *t*_r-f_ values of 400 ns, 200 ns, and 100 ns; the point ① is the midpoint of the low level of the pulse, and the points ②, ③, and ④ are the beginning, midpoint, and end of the high-level duration, respectively. At the point ①, a relatively weak potential is formed across the channel and dielectric layers, and there is no significant difference in the potential distributions for *t*_r-f_ values of 400 ns, 200 ns, and 100 ns. At point ② where the AC voltage reaches 15 V, a strong potential is formed across the dielectric and channel. When the AC voltage rapidly increases (*t*_r-f_ is 100 ns), a wide and strong potential forms across the dielectric and channel layers, which is related to the current overshoot phenomenon during the pulse rising edge. This wide and strong potential at fast rising times allows more electrons to be injected into the dielectric. Consequently, a higher gate leakage current (*I*_GS_) is observed at a faster *t*_r-f_. On the other hand, at the points ③ and ④, the potential in the channel becomes weak regardless of the *t*_r-f_. Figure 5b shows the *I*_GS_ during one cycle of an AC pulse with *t*_r-f_ values of 400 ns, 200 ns, and 100 ns. Compared to *I*_DS_, which is commonly used to evaluate the electrical performance and degradation behavior of TFTs, *I*_GS_ is an appropriate parameter to find out the cause of *V*_TH_ degradation using the rising time of the AC bias. The extracted *I*_GS_ values at the end of the rising edge are 0.18 mA, 0.24 mA, and 0.33 mA, respectively. The magnitude of the current overshoot increases as *t*_r-f_ decreases. Because the current overshoot can cause significant degradation in electronic device performance [31,32], it is reasonable to attribute the increase in Δ*V*_TH_ to the faster rising time. Therefore, at fast rising times, the wide and strong potential formed across the dielectric and channel layers leads to an increased Δ*V*_TH_.

Additionally, this study reveals that *I*_GS_ is a key parameter to analyze the current overshoot phenomenon occurring when the AC bias stress is applied to the gate electrode.

**Figure 6:**
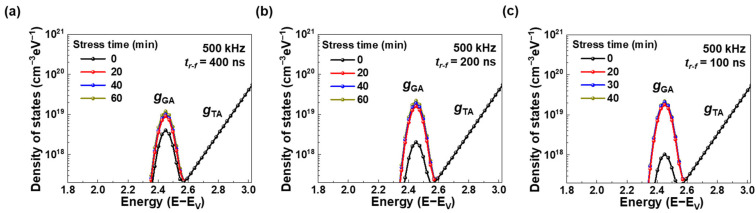
Density of states in the a-ITGZO TFTs under AC bias stress with *t*_r-f_ of (**a**) 400 ns, (**b**) 200 ns, and (**c**) 100 ns.

On the other hand, electron trapping at the interface traps was also examined by analyzing the density of states of a-ITGZO TFTs under AC bias stress with *t*_r-f_ values of 400 ns (Figure 6a), 200 ns (Figure 6b), and 100 ns (Figure 6c), as shown in Figure 6.

The distributions of the sub-gap trap states for oxide semiconductors were calculated as follows:

(4) $$g_{TA}\left(E\right)=N_{TA}\;exp\left[\frac{E-E_{C}}{W_{TA}}\right]$$

(5) $$g_{TD}\left(E\right)=N_{TD}\;exp\left[\frac{E_{V}-E}{W_{TD}}\right]$$

(6) $$g_{GA}\left(E\right)=N_{GA}\;exp\left[-{\left(\frac{E_{GA}-E}{W_{GA}}\right)}^{2}\right]$$

(7) $$g_{GD}\left(E\right)=N_{GD}\;exp\left[-{\left(\frac{{E-E}_{GD}}{W_{GD}}\right)}^{2}\right]$$

where *g*_TA_(E), *g*_TD_(E), *g*_GA_(E), and *g*_GD_(E) represent the acceptor-like tail states, donor-like tail states, acceptor-like Gaussian states, and donor-like Gaussian states, respectively. The parameters *N*_TD_ and *N*_TA_ indicate the effective state densities at the valence band maximum (*E*_V_) and conduction band minimum (*E*_C_), while *W*_TD_ and *W*_TA_ correspond to the characteristic decay slopes of the tail states near the respective band edges. In addition, the parameters *N*_GD_ and *N*_GA_ refer to the total concentration of Gaussian-distributed donor-like and acceptor-like states, respectively. Their corresponding peak energies are denoted as *E*_GD_ and *E*_GA_, while *W*_GD_ and *W*_GA_ represent the characteristic decay energy. There are no changes in *g*_TD_(E), *g*_GD_(E), or *g*_TA_(E), while *g*_GA_(E) increases after AC bias stress. Specifically, the increase in *g*_GA_(E) becomes more significant as the pulse *t*_r-f_ becomes faster. The *N*_GA_ standing for the interface trap density increases from 4.0 × 10^18^ cm^−3^eV^−1^ to 1.2 × 10^19^ cm^−3^eV^−1^ for *t*_r-f_ = 400 ns, from 2.0 × 10^18^ cm^−3^eV^−1^ to 2.2 × 10^19^ cm^−3^eV^−1^ for *t*_r-f_ = 200 ns, and from 1.0 × 10^18^ cm^−3^eV^−1^ to 2.2 × 10^19^ cm^−3^eV^−1^ for *t*_r-f_ = 100 ns. The interface trap density significantly increases when the AC pulse has a short rise time. According to previous studies [33,34,35], weakly bonded oxygen ions are easily ionized under AC bias stress due to their low formation energy, forming oxygen interstitials that act as *g*_GA_(E) states at the interface between the channel and dielectric layers. Hence, an AC pulse with a faster *t*_r-f_ accelerates the electron trapping of *g*_GA_(E) during bias stress, resulting in a larger Δ*V*_TH_. This is consistent with the stretched exponential model described above. In this study, it was revealed that AC stress-induced performance degradation in a-ITGZO TFTs is attributed to electron trapping both in the bulk states of the gate dielectric and at the interface traps between the channel and dielectric layers.

## 4. Conclusions

In this study, we investigated the effect of the pulse rising time on AC stress-induced performance degradation in a-ITGZO TFTs through both experiments and simulations. When AC bias stress was applied with *t*_r-f_ values of 400 ns, 200 ns, and 100 ns, *V*_TH_ was positively shifted by 0.97 V, 2.68 V, and 2.83 V, respectively. However, the on-current and *SS* of the a-ITGZO TFTs under AC bias stress showed little change compared to those of the pristine devices. Accordingly, a stretched exponential model was applied to describe Δ*V*_TH_. Based on the potential distribution formed across the dielectric and channel layers as a function of *t*_r-f_, it was confirmed that electron trapping in the bulk states of the gate dielectric contributes to the observed Δ*V*_TH_. Additionally, the larger Δ*V*_TH_ under faster *t*_r-f_ conditions was attributed to the electron trapping of *g*_GA_(E) interface states during bias stress. Overall, this study demonstrates that the degradation in Δ*V*_TH_ of oxide TFTs under AC bias stress results from both electron trapping in the bulk states of the gate dielectric and trapping at the interface traps between the channel and dielectric layers.

## Data Availability

Data is contained within the article.
